# A reliable algorithm to compute the approximate solution of KdV-type partial differential equations of order seven

**DOI:** 10.1371/journal.pone.0244027

**Published:** 2021-01-22

**Authors:** Sidra Saleem, Malik Zawwar Hussain, Imran Aziz

**Affiliations:** 1 Department of Mathematics, University of the Punjab, Lahore, Pakistan; 2 Department of Mathematics, University of Peshawar, Peshawar, Pakistan; China University of Mining and Technology, CHINA

## Abstract

The approximate solution of KdV-type partial differential equations of order seven is presented. The algorithm based on one-dimensional Haar wavelet collocation method is adapted for this purpose. One-dimensional Haar wavelet collocation method is verified on Lax equation, Sawada-Kotera-Ito equation and Kaup-Kuperschmidt equation of order seven. The approximated results are displayed by means of tables (consisting point wise errors and maximum absolute errors) to measure the accuracy and proficiency of the scheme in a few number of grid points. Moreover, the approximate solutions and exact solutions are compared graphically, that represent a close match between the two solutions and confirm the adequate behavior of the proposed method.

## 1 Introduction

The importance of the nonlinear phenomena of differential equations (DEs) in sciences (biology, physics and chemistry) is significant. In any branch of natural sciences, there exists a small number of problems, that can be solved in a direct way. So, to understand the complete physical phenomena, the mathematical modeling comes into account by means of partial differential equations (PDEs), that shows an exceptional performance in science and engineering. The concept of the physical phenomena, depends upon the nature of the solutions of PDEs, so to find the solutions of PDEs, the analytical, semi analytical and numerical methods are introduced. It has been a requirement to choose a competent mechanism that adopts such mathematical models which cover physical processes as well [1].

In nonlinear sciences and engineering, analytical solutions to nonlinear PDEs have a great significance but sometimes, it becomes much problematic to deal with, that becomes a hurdle in finding analytical solutions to these DEs. So, the scientists have introduced many techniques like perturbation methods, approximation methods and numerical methods to overcome the problems of such DEs. Considering the effect of nonlinearity, many analytical techniques concentrate on linearizing the physical phenomena, that yields the variation in the nature of these analytical solutions from the actual ones and becomes a cause of change in the real physics of the given phenomena [1].

Several efforts have been exerted for finding and improving the dynamic, robust and effective schemes to solve the nonlinear PDEs (KdV equation, Burgers’ equation, KdV Burgers’ equation, Helmholtz equation, Schrödinger equation, Klein-Gordon equation, diffusion equation, sine-Gordon equation and Fisher’s equation), for example inverse scattering scheme, homotopy perturbation method, tanh-sech method, F-expansion method, sine-cosine method, tanh-function method, Hirota’s bilinear method, exp-function method, Jacobi elliptic functions method, (G/G’)-expansion method, Riemann-Hilbert approach and many more [2, 3–10].

Kortweg-de Vries (KdV) equation is a nonlinear PDE, that is used to model travelling waves in shallow water and harmonic crystal. KdV was proposed by Boussinesq about 1877 and determined by Kortweg-de Vries about 1895. Moreover, the well known KdV equation of order seven was introduced by Pomeau *et al*. [11] in a research paper, to study its stability underneath a singular (restricted) perturbation. These days, the development of KdV equation is important in the phenomena of fluid dynamics, magma flow, conduit waves, optical fibers, waves in plasma physics and flow in blood vessels. The significance of KdV does not exist in their utilization only but also in some sort of belongings that are not normally anticipated by nonlinear PDEs. Moreover, KdV can have multi-soliton solutions along solitary wave solutions, when these interesting facts about KdV had been introduced, many authors presented the initial value problem associated to the KdV from theoretical and numerical point of view [12, 13].

In literature, there exist many analytical and numerical schemes like He’s variation iteration method, pseudospectral method, Adomian decomposition method (ADM), Bäcklund transformations to solve such problems, finite difference method (FDM), finite element method (FEM), finite volume method (FVM), homotopy analysis method (HAM), Fourier spectral method (FSM) and variation iteration method (VIM) to solve such problems [1, 2, 14–17], some of them are given by:

Arora and Sharma [1] approximated the seventh order KdV equations for example Sawada-Kotera-Ito equation, Lax equation and Kaup-Kuperschmidt equation by HAM. They used a parameter namely *h* to control the convergence of the method and by fixing it, the computational results were compared with the analytical solutions. That indicated the robustness and elegance behavior of the proposed scheme.

Darvishi *et al*. [2] solved the Lax’s seventh-order KdV equation by pseudospectral method. Darvishi’s preconditioning was used for the accuracy of matrix vector multiplication. There was a good agreement observed between numerical solution and exact solution. Saravi *et al*. [14] reconstructed the variational iteration method (VIM) for the numerical solution of Lax’s and Sawada-Kotera equations of order seven. They compared the results with the Adomian decomposition method (ADM) and existing analytical solutions. The computational outcomes indicated the effectiveness of VIM for both equations.

Aljahdaly [18] presented two applications of Lax-equation and Kaup-Kuperschmidt equation of order seven by constructing new travelling wave solutions. Moreover, nonlinear PDEs were solved analytically by modified auxiliary equation, that resulted the stable analytical solutions. Ganji and Abdollahzadeh [19] solved a nonlinear evolution equation analytically. The sech-method and the exp-function method were used to form the solitary travelling wave solutions of Lax’s (KdV) equation of order seven, that were important from physical point of view.

Sharma and Arora [20] established the modified form of He’s variational iteration method (MVIM) to solve some non-linear partial differential equations analytically, such as Fisher-type equation, cubic Boussinesq equation and Caudrey-Dodd-Gibbon equation of order seven. The numerical results indicated the efficient behavior of the prescribed method. The strength of MVIM over standard form of VIM was investigated, as it assured less computational cost. Salas implemented Cole-Hopf transform for construction of analytical solutions for generalization of Lax’s KdV equation and the Sawada-Kotera-Ito KdV equation of order seven with forcing term [21].

Since the frequently used techniques involve FDM, FEM, FVM and FSM for the approximation of DEs. Along with many benefits these numerical methods were facing some deficiencies, that encouraged the mathematicians to do struggle for more better numerical schemes and a wide range of numerical schemes including wavelet schemes were introduced. The wavelet techniques are widely used to avoid the complications of science and engineering [22, 23] and advantageous over FDM, FEM, FVM and FSM.

The word wavelet was coined by Morlet and Grossmann [24] and the analysis about wavelets was presented by Morlet, Grossmann and Meyer and gained a great success in the field of wavelets [24, 25]. Mallat gave the idea of multiresolution analysis (MRA), that used to provide high resolution for simulation of nonlinear and singular equations [26]. In 1988, Daubechies introduced a scheme with wavelets having compact support and scaling functions [26]. The appealing features of wavelet methods include coping with the singularities, rough structures and unstable phenomena, that are exhibited by the analyzed equations. In addition, for the numerical solution of PDEs wavelets are established on collocation procedure and Galerkin techniques.

For approximation of PDEs, the authors have used many wavelet techniques but Haar wavelet (HW) is considered simple and efficient among them. Many models in different eras of science and engineering are constructed with the aid of HW [27]. Alfred Haar was the founder of HW, he introduced it in 1909 [28].

Haar functions are built up on piecewise constant functions with orthonormal basis. Due to their behavior of compact support, they become zero after a finite interval. So, being local they can easily handle irregularities, that’s why HW is considered preferable over other wavelets. In addition, HW is a special case of wavelet introduced by Daubechies [29].

The derivatives of HW functions fail to exist at the points of discontinuities, that become the reason of failure of applying HW directly to solve PDEs, to sort out this difficulty, the integration of wavelets has been introduced [27]. The techniques of HW functions have been tested in different fields like to denoise the noisy data, for analyzing the time frequency and for approximation of linear and nonlinear differential, integro-differential and integral equations [30–42].

Aziz *et al*. introduced quadrature rules for the of double and triple integrals on the basis of HW and compound functions. The presented technique was observed more accurate and flexible in comparison with the techniques existing in the literature by finding absolute errors [43].

Lepik solved nonlinear evolution equations numerically by a scheme based on HW functions. The author applied HW method on Burgers’ and sine-Gordon equations and compared the obtained and already existing results in the literature. It was noticed, that the proposed method was computationally economical [44]. The general form of seventh-order KdV-type equations is given by [18, 45]:

$$\begin{array}{c} u_{t}+\alpha u^{3}u_{x}+\beta u_{x}^{3}+\gamma uu_{x}u_{2x}+\mu u^{2}u_{3x}+\nu u_{2x}u_{3x}+\phi fu_{x}u_{4x}+\psi uu_{5x}+u_{7x}=0, \end{array}$$ (1)

where *α*, *β*, *γ*, *μ*, *ν*, *ϕ* and *ψ* are constants subject to initial condition(IC):

$$\begin{array}{c} u(x,0)=f(x), \end{array}$$ (2)

and boundary conditions(BCs):

$$\begin{array}{c} \begin{array}{cc} & u\left(0,t\right)=g_{1}\left(t\right),\;u\left(1,t\right)=g_{2}\left(t\right),\;u_{x}\left(0,t\right)=g_{3}\left(t\right),\;u_{x}\left(1,t\right)=g_{4}\left(t\right),\\ & u_{2x}\left(0,t\right)=g_{5}\left(t\right),\;u_{2x}\left(1,t\right)=g_{6}\left(t\right),\;u_{3x}\left(0,t\right)=g_{7}\left(t\right). \end{array} \end{array}$$ (3)

This form is the universal model for the study of shallow water waves along-with surface tension and magneto-acoustic waves in plasma. In the current study, we will explore the special types of this equation which are given below:

For *α* = 140, *β* = 70, *γ* = 280, *μ* = 70, *ν* = 70, *ϕ* = 42 and *ψ* = 14, it is called Lax equation of order seven:

$$\begin{array}{c} u_{t}+140u^{3}u_{x}+70u_{x}^{3}+280uu_{x}u_{2x}+70u^{2}u_{3x}+70u_{2x}u_{3x}+42u_{x}u_{4x}+14uu_{5x}+u_{7x}=0. \end{array}$$ (4)

For *α* = 252, *β* = 63, *γ* = 378, *μ* = 126, *ν* = 63, *ϕ* = 42 and *ψ* = 21, it is called seventh-order Sawada-Kotera (SK) equation:

$$\begin{array}{c} u_{t}+252u^{3}u_{x}+63u_{x}^{3}+378uu_{x}u_{2x}+126u^{2}u_{3x}+63u_{2x}u_{3x}+42u_{x}u_{4x}+21uu_{5x}+u_{7x}=0. \end{array}$$ (5)

For *α* = 2016, *β* = 630, *γ* = 2268, *μ* = 504, *ν* = 252, *ϕ* = 147 and *ψ* = 42, it is called Kaup-Kuperschmidt (KK) equation of order seven:

$$\begin{array}{c} u_{t}+2016u^{3}u_{x}+630u_{x}^{3}+2268uu_{x}u_{2x}+504u^{2}u_{3x}+252u_{2x}u_{3x}+147u_{x}u_{4x}+42uu_{5x}+u_{7x}=0. \end{array}$$ (6)

The seventh order KdV-type equations are utilized to study different nonlinear phenomena of physical situations and its role is significant in the phenomena of wave propagations [46].

The aim of this research work is to choose an authentic approximation of seventh-order KdV-type equations (Lax, Sawada-Kotera-Ito and Kaup-Kuperschmidt equations) using one-dimensional Haar wavelet collocation method (1-D HWCM), that provides high quality computed results in short time with a few grid points.

The proposed research work is a part of thesis [47] and it is arranged in the subsequential style. In Section 2, the definitions of MRA and HW are given. In Section 3, the proposed numerical method for seventh-order KdV-type equations is demonstrated. In Section 4, the convergence theorem of the HW is discussed. The computed results are reported in Section 5. Finally, in Section 6, a few concluding remarks of the research work are presented.

## 2 Materials and methods

### 2.1 Multi-resolution analysis

The concept of wavelets becomes clear via multi-resolution analysis (MRA), where *f* is considered a function from the class of all square integrable functions over the real line, doing MRA of *L*_2_(*R*), ${\tilde{\chi }}_{\tilde{m}},{\tilde{\chi }}_{\tilde{m}+1},\ldots$ of subspaces can be generated in a way that the projection of *f* onto these spaces gives finer approximations of the function *f* as $\tilde{m}\to \infty .$ MRA of *L*_2_(*R*) yields ${\tilde{\chi }}_{\tilde{m}}\subset L_{2}\left(R\right)$, $\tilde{m}\in Z$ that is a sequence of closed subspaces, with the following properties:

- Monotonicity $\cdots \subset {\tilde{\chi }}_{-1}\subset {\tilde{\chi }}_{0}\subset {\tilde{\chi }}_{1}\subset \ldots$
- ${\tilde{\chi }}_{\tilde{m}}\text{s}$ fulfill the condition that ${⋃}_{\tilde{m}\in Z}{\tilde{\chi }}_{\tilde{m}}$ is dense in *L*_2_(*R*) and ${⋂}_{\tilde{m}\in Z}{\tilde{\chi }}_{\tilde{m}}=0$.
- If $f\left(x\right)\in {\tilde{\chi }}_{0}$, then $f\left(2^{\tilde{m}}x\right)\in {\tilde{\chi }}_{\tilde{m}}$. In other words, this statement indicates that ${\tilde{\chi }}_{\tilde{m}}s$ can be obtained from the central space ${\tilde{\chi }}_{0}$ by the process of scaling.
- If $f\left(x\right)\in {\tilde{\chi }}_{0}$, then $f\left(2^{\tilde{m}}x-\tilde{n}\right)\in {\tilde{\chi }}_{\tilde{m}}$. In other words, ${\tilde{\chi }}_{\tilde{m}}s$ remain unchanged when the process of translation is applied to them.
- A function $\tilde{\phi }\in {\tilde{\chi }}_{0}$ is obtained in a way that the set $\left\{\tilde{\phi }\left(x-\tilde{n}\right):\tilde{n}\in Z\right\}$ forms a Riesz basis in ${\tilde{\chi }}_{0}$.

The approximation of general functions is performed with the help of the space ${\tilde{\chi }}_{\tilde{m}}$ by defining proper projection (of these functions). Any square integrable function in *L*_2_(*R*) may be approximated arbitrarily closed by using these projections due to the property, that is the union of ${\tilde{\chi }}_{\tilde{m}}\text{s}$ is closed in *L*_2_(*R*). For instance, if the space ${\tilde{\chi }}_{\tilde{m}}$ is defined as:

$$\begin{array}{c} {\tilde{\chi }}_{\tilde{m}}={\tilde{\varrho }}_{\tilde{m}-1}⊕{\tilde{\chi }}_{\tilde{m}-1}={\tilde{\varrho }}_{\tilde{m}-1}⊕{\tilde{\varrho }}_{\tilde{m}-2}⊕{\tilde{\chi }}_{\tilde{m}-2}=\overset{\tilde{m}+1}{\underset{\tilde{m}=1}{⊕}}{\tilde{\varrho }}_{\tilde{m}}⊕{\tilde{\varrho }}_{0}, \end{array}$$ (7)

then after translation and dilation, MRA may be formed for the sequence of spaces $\left\{{\tilde{\chi }}_{\tilde{m}},\tilde{m}\in Z\right\}$ given by Eqs (8) and (10) by using the scaling function *ς*_1_(*x*). It is noticeable, that for all $\tilde{m}$, the space ${\tilde{\varrho }}_{\tilde{m}}$ is an orthogonal complement of ${\tilde{\chi }}_{\tilde{m}}$ in ${\tilde{\chi }}_{\tilde{m}}+1$, which means that w.r.t. any given inner product, the space ${\tilde{\varrho }}_{\tilde{m}}$ contains all those functions in ${\tilde{\chi }}_{\tilde{m}}+1$ which are orthogonal to all those in ${\tilde{\chi }}_{\tilde{m}}$. The basis which is formed by the set of functions for the space ${\tilde{\varrho }}_{\tilde{m}}$ is called a formation of wavelets [26].

### 2.2 Haar wavelet

The HW functions are the functions with compact support, they are based on bounded intervals and are defined on [0, 1). These functions (except Haar scaling function) are with unique representation given as [27]:

$$\begin{array}{c} \varsigma_{\tilde{l}}\left(x\right)=\{\begin{array}{cc} 1, & \forall \;x\in [\tilde{o},\tilde{p})\\ -1, & \forall \;x\in \left[\tilde{p},\tilde{q}\right),\;\;\;\tilde{l}=2,3,\cdots \\ 0, & \;\text{elsewhere} \end{array} \end{array}$$ (8)

with

$$\begin{array}{c} \tilde{o}=\frac{\tilde{n}}{\tilde{r}},\;\tilde{p}=\frac{\tilde{n}+0.5}{\tilde{r}},\;\tilde{q}=\frac{\tilde{n}+1}{\tilde{r}}, \end{array}$$ (9)

where $\tilde{l}$ is used for wavelet number, $\tilde{o},\tilde{p}$ and $\tilde{q}$ are constants, $\tilde{n}$ is the translation parameter and $\tilde{r}$ is used for determining the resolution level.

For approximation by HW functions, MRA performs a great role, here, $\tilde{M}$ is used for maximum level of resolution, whereas, the level of HW is taken as the integral values of $\tilde{m}$ with $\tilde{m}=log_{2}\tilde{r}$, that implies $\tilde{r}=2^{\tilde{m}}$ with $\tilde{m}=0,1,2,...,\tilde{M}$. Moreover, the wavelet number $\tilde{l}$ satisfies the relation $\tilde{l}=\tilde{r}+\tilde{n}+1$, by taking $\tilde{l}=2$, we are with *ς*_2_(*x*), that is mother wavelet (HW function). The father wavelet (Haar scaling function) *ς*_1_(*x*), has the following representation:

$$\begin{array}{c} \varsigma_{1}\left(x\right)=\{\begin{array}{cc} 1, & \forall \;x\in [0,1)\\ 0, & \text{elsewhere} \end{array} \end{array}$$ (10)

Any member of the family of square integrable functions defined on [0, 1) can be represented as an infinite sum of HW functions as:

$$\begin{array}{c} f\left(x\right)=\sum_{\tilde{l}=1}^{\infty }\;{\overset{⌄}{a}}_{\tilde{l}}\varsigma_{\tilde{l}}\left(x\right). \end{array}$$ (11)

We identify the integer $\tilde{R}=2^{\tilde{M}}$ and $\tilde{S}=2\tilde{R}=2^{\tilde{M}+1}$, to define discrete HW functions, where $\tilde{M}$ is maximum resolution level. So, any function *f*(*x*) on [0, 1) can be estimated as a finite sum of HW functions in the following manner:

$$\begin{array}{c} f\left(x\right)=\sum_{\tilde{l}=1}^{\tilde{S}}\;{\overset{⌄}{a}}_{\tilde{l}}\varsigma_{\tilde{l}}\left(x\right). \end{array}$$ (12)

The concept of Haar integrals were derived by Chen and Hsiao in 1997 [48], here, the Haar integrals are presented by:

$$\begin{array}{c} \delta_{\tilde{l},1}\left(x\right)=\int_{0}^{x}\varsigma_{\tilde{l}}\left(x^{\prime }\right)dx^{\prime }, \end{array}$$

$$\begin{array}{c} \delta_{\tilde{l},\;\kappa +1}\left(x\right)=\int_{0}^{x}\varsigma_{\tilde{l},\kappa }\left(x^{\prime }\right)dx^{\prime },\;\;\;\;\kappa =1,2,\ldots \end{array}$$

We can calculate these integrals using Eq (8) and the first two integrals are given below:

$$\begin{array}{c} \delta_{\tilde{l},1}\left(x\right)=\{\begin{array}{cc} x-\tilde{o}, & \forall \;x\in [\tilde{o},\tilde{p})\\ \tilde{q}-x, & \forall \;x\in [\tilde{p},\tilde{q})\\ 0, & otherwise \end{array} \end{array}$$ (13)

$$\begin{array}{c} \delta_{\tilde{l},2}\left(x\right)=\{\begin{array}{cc} \frac{1}{2}{\left(x-\tilde{o}\right)}^{2}, & \forall \;x\in [\tilde{o},\tilde{p})\\ \frac{1}{2}{\left(\tilde{q}-x\right)}^{2}, & \forall \;x\in [\tilde{p},\tilde{q})\\ 0, & otherwise \end{array} \end{array}$$ (14)

The general expression for the Haar integrals is given by

$$\begin{array}{c} \delta_{\tilde{l},\tilde{s}}\left(x\right)=\{\begin{array}{cc} 0, & \forall \;x\in [0,\tilde{o})\\ \frac{1}{\tilde{s}!}{\left(x-\tilde{o}\right)}^{\tilde{s}}, & \forall \;x\in [\tilde{o},\tilde{p})\\ \frac{1}{\tilde{s}!}[{\left(x-\tilde{o}\right)}^{\tilde{s}}-2{\left(x-\tilde{p}\right)}^{\tilde{s}}], & \forall \;x\in [\tilde{p},\tilde{q})\\ \frac{1}{\tilde{s}!}[{\left(x-\tilde{o}\right)}^{\tilde{s}}-2{\left(x-\tilde{p}\right)}^{\tilde{s}}+{\left(x-\tilde{q}\right)}^{\tilde{s}}], & \forall \;x\in [\tilde{q},1) \end{array} \end{array}$$ (15)

The above expression $\delta_{\tilde{l},\tilde{s}}\left(x\right)$ is for $\tilde{l}=2,3,\ldots$ and $\tilde{s}=1,2,\ldots$, for $\tilde{l}=1$ and $\tilde{s}=1,2,\ldots$, we have $\delta_{\tilde{l},\tilde{s}}\left(x\right)=\frac{x^{\tilde{s}}}{\tilde{s}!}$.

## 3 The proposed approximation method

The presented technique is constructed on the basis of FDM and 1-D HWCM for Eq (1). The space derivatives in Eq (1) are discretized using 1-D HWCM, whereas, the discretization of the temporal derivative in Eq (1) is enforced using FDM, that is:

$$\begin{array}{c} \frac{\partial u}{\partial t}\approx \frac{u^{\tilde{s}+1}-u^{\tilde{s}}}{\Delta t}, \end{array}$$ (16)

where $u^{\tilde{s}}=u\left(x,t_{\tilde{s}}\right)$, $t_{\tilde{s}+1}=t_{\tilde{s}}+\Delta t$, $\tilde{s}=0,1,\ldots ,Ϝ/\Delta t$ and *t*_0_ = 0. Here, we assume that *x* ∈ Ψ = [0, 1], *t* ∈ ∂Ψ. For our proposed method, we consider the collocation points (CPs) as:

$$\begin{array}{c} x_{\tilde{m}}=\frac{\tilde{m}-0.5}{\tilde{S}},\;\tilde{m}=1,2,\ldots \tilde{S}, \end{array}$$

$$\begin{array}{c} t_{\tilde{m}}=\frac{\tilde{m}-0.5}{\tilde{S}},\;\tilde{m}=1,2,\ldots \tilde{S}. \end{array}$$

Now, applying finite difference scheme to the KdV Eq (1)

$$\begin{array}{c} \begin{array}{cc} & \frac{u^{\tilde{s}+1}-u^{\tilde{s}}}{\Delta t}+\alpha u^{3(\tilde{s}+1)}u_{x}^{\tilde{s}+1}+\beta u_{x}^{3(\tilde{s}+1)}+\gamma u^{\tilde{s}+1}u_{x}^{\tilde{s}+1}u_{2x}^{\tilde{s}+1}+\mu u^{2(\tilde{s}+1)}u_{3x}^{\tilde{s}+1}+\nu u_{2x}^{\tilde{s}+1}u_{3x}^{\tilde{s}+1}\\ & +\phi u_{x}^{\tilde{s}+1}u_{4x}^{\tilde{s}+1}+\psi u^{\tilde{s}+1}u_{5x}^{\tilde{s}+1}+u_{7x}^{\tilde{s}+1}=0. \end{array} \end{array}$$ (17)

Linearizing the nonlinear terms of Eq (17), we have

$$\begin{array}{c} \begin{array}{cc} & \alpha \Delta t(u^{3\tilde{s}}u_{x}^{\tilde{s}+1}+3u^{2\tilde{s}}u^{\tilde{s}+1}u_{x}^{\tilde{s}}-3u^{3\tilde{s}}u_{x}^{\tilde{s}})+\beta \Delta t(3u_{x}^{\tilde{s}+1}u_{x}^{2\tilde{s}}-2u_{x}^{3\tilde{s}})\\ & +\psi \Delta t(u^{\tilde{s}}u_{5x}^{\tilde{s}+1}+u^{\tilde{s}+1}u_{5x}^{\tilde{s}}-u^{\tilde{s}}u_{5x}^{\tilde{s}})\\ & +\gamma \Delta t(u^{\tilde{s}}u_{x}^{\tilde{s}+1}u_{2x}^{\tilde{s}}-2u^{\tilde{s}}u_{x}^{\tilde{s}}u_{2x}^{\tilde{s}}+u_{x}^{\tilde{s}}u^{\tilde{s}}u_{2x}^{\tilde{s}+1}+u^{\tilde{s}+1}u_{x}^{\tilde{s}}u_{2x}^{\tilde{s}})\\ & +\mu \Delta t(u_{3x}^{\tilde{s}+1}u^{2\tilde{s}}+2u^{\tilde{s}+1}u^{\tilde{s}}u_{3x}^{\tilde{s}+1}-2u^{2\tilde{s}}u_{3x}^{\tilde{s}})+u^{\tilde{s}+1}-u^{\tilde{s}}\\ & +\nu \Delta t(u_{2x}^{\tilde{s}}u_{3x}^{\tilde{s}+1}+u_{2x}^{\tilde{s}+1}u_{3x}^{\tilde{s}}-u_{2x}^{\tilde{s}}u_{3x}^{\tilde{s}})+\Delta tu_{7x}^{\tilde{s}+1}\\ & +\phi \Delta t(u_{x}^{\tilde{s}}u_{4x}^{\tilde{s}+1}+u_{x}^{\tilde{s}+1}u_{4x}^{\tilde{s}}-u_{x}^{\tilde{s}}u_{4x}^{\tilde{s}})=0. \end{array} \end{array}$$ (18)

After simplifications, we have

$$\begin{array}{c} \begin{array}{cc} & (1+\Delta t(\gamma u_{x}^{\tilde{s}}u_{2x}^{\tilde{s}}+2\mu u^{\tilde{s}}u_{3x}^{\tilde{s}}+\psi tu_{5x}^{\tilde{s}}+3\alpha u^{2\tilde{s}}u_{x}^{\tilde{s}}))u^{\tilde{s}+1}\\ & +\Delta t(\alpha u^{3\tilde{s}}+3\beta u_{x}^{2\tilde{s}}+\gamma u^{\tilde{s}}u_{2x}^{\tilde{s}}+\phi u_{4x}^{\tilde{s}})u_{x}^{\tilde{s}+1}+\Delta t(\gamma u_{x}^{\tilde{s}}u^{\tilde{s}}+\nu u_{3x}^{\tilde{s}})u_{2x}^{\tilde{s}+1}\\ & +\Delta t(\mu u^{2\tilde{s}}+\nu u_{2x}^{\tilde{s}})u_{3x}^{\tilde{s}+1}+\Delta t\phi u_{x}^{\tilde{s}}u_{4x}^{\tilde{s}+1}+\Delta t\psi u^{\tilde{s}}u_{5x}^{\tilde{s}+1}+\Delta tu_{7x}^{\tilde{s}+1}\\ & =u^{\tilde{s}}+3\alpha \Delta tu^{3\tilde{s}}u_{x}^{\tilde{s}}+2\beta \Delta tu_{x}^{3\tilde{s}}+2\gamma \Delta tu^{\tilde{s}}u_{x}^{\tilde{s}}u_{2x}^{\tilde{s}}+2\mu \Delta tu^{2\tilde{s}}u_{3x}^{\tilde{s}}\\ & +\nu \Delta tu_{2x}^{\tilde{s}}u_{3x}^{\tilde{s}}+\phi \Delta tu_{x}^{\tilde{s}}u_{4x}^{\tilde{s}}+\psi \Delta tu^{\tilde{s}}u_{5x}^{\tilde{s}}, \end{array} \end{array}$$ (19)

where the nonlinear terms,

$$\begin{array}{c} u^{3(\tilde{s}+1)}u_{x}^{\tilde{s}+1},\;u_{x}^{3(\tilde{s}+1)},\;u^{\tilde{s}+1}u_{x}^{\tilde{s}+1}u_{2x}^{\tilde{s}+1},\;u^{2(\tilde{s}+1)}u_{3x}^{\tilde{s}+1},\;u_{2x}^{\tilde{s}+1}u_{3x}^{\tilde{s}+1},\;u_{x}^{\tilde{s}+1}u_{4x}^{\tilde{s}+1},\;u^{\tilde{s}+1}u_{5x}^{\tilde{s}+1} \end{array}$$

are linearized by applying quasi-Newton linearization technique as follows:

$$\begin{array}{c} u^{3(\tilde{s}+1)}u_{x}^{\tilde{s}+1}=u^{3\tilde{s}}u_{x}^{\tilde{s}+1}+3u^{2\tilde{s}}u^{\tilde{s}+1}u_{x}^{\tilde{s}}-3u^{3\tilde{s}}u_{x}^{\tilde{s}}, \end{array}$$

$$\begin{array}{c} u_{x}^{3(\tilde{s}+1)}=3u_{x}^{\tilde{s}+1}u_{x}^{2\tilde{s}}-2u_{x}^{3\tilde{s}}, \end{array}$$

$$\begin{array}{c} u^{\tilde{s}+1}u_{x}^{\tilde{s}+1}u_{2x}^{\tilde{s}+1}=u^{\tilde{s}}u_{x}^{\tilde{s}+1}u_{2x}^{\tilde{s}}-2u^{\tilde{s}}u_{x}^{\tilde{s}}u_{2x}^{\tilde{s}}+u_{x}^{\tilde{s}}u^{\tilde{s}}u_{2x}^{\tilde{s}+1}+u^{\tilde{s}+1}u_{x}^{\tilde{s}}u_{2x}^{\tilde{s}}, \end{array}$$

$$\begin{array}{c} u^{2(\tilde{s}+1)}u_{3x}^{\tilde{s}+1}=u_{3x}^{\tilde{s}+1}u^{2\tilde{s}}+2u^{\tilde{s}+1}u^{\tilde{s}}u_{3x}^{\tilde{s}}-2u^{2\tilde{s}}u_{3x}^{\tilde{s}}, \end{array}$$

$$\begin{array}{c} u_{2x}^{\tilde{s}+1}u_{3x}^{\tilde{s}+1}=u_{2x}^{\tilde{s}}u_{3x}^{\tilde{s}+1}+u_{2x}^{\tilde{s}+1}u_{3x}^{\tilde{s}}-u_{2x}^{\tilde{s}}u_{3x}^{\tilde{s}}, \end{array}$$

$$\begin{array}{c} u_{x}^{\tilde{s}+1}u_{4x}^{\tilde{s}+1}=u_{x}^{\tilde{s}}u_{4x}^{\tilde{s}+1}+u_{x}^{\tilde{s}+1}u_{4x}^{\tilde{s}}-u_{x}^{\tilde{s}}u_{4x}^{\tilde{s}}, \end{array}$$

$$\begin{array}{c} u^{\tilde{s}+1}u_{5x}^{\tilde{s}+1}=u^{\tilde{s}}u_{5x}^{\tilde{s}+1}+u^{\tilde{s}+1}u_{5x}^{\tilde{s}}-u^{\tilde{s}}u_{5x}^{\tilde{s}}. \end{array}$$

Now applying CPs, we have

$$\begin{array}{c} \begin{array}{cc} & (1+\Delta t(\gamma u_{x_{k}}^{\tilde{s}}u_{2x_{k}}^{\tilde{s}}+2\mu u^{\tilde{s}}u_{3x_{k}}^{\tilde{s}}+\psi tu_{5x_{k}}^{\tilde{s}}+3\alpha u^{2\tilde{s}}u_{x_{k}}^{\tilde{s}}))u^{\tilde{s}+1}\\ & +\Delta t(\alpha u^{3\tilde{s}}+3\beta u_{x_{k}}^{2\tilde{s}}+\gamma u^{\tilde{s}}u_{2x_{k}}^{\tilde{s}}+\phi u_{4x_{k}}^{\tilde{s}})u_{x_{k}}^{\tilde{s}+1}+\Delta t(\gamma u_{x_{k}}^{\tilde{s}}u^{\tilde{s}}+\nu u_{3x_{k}}^{\tilde{s}})u_{2x_{k}}^{\tilde{s}+1}\\ & +\Delta t(\mu u^{2\tilde{s}}+\nu u_{2x_{k}}^{\tilde{s}})u_{3x_{k}}^{\tilde{s}+1}+\Delta t\phi u_{x_{k}}^{\tilde{s}}u_{4x_{k}}^{\tilde{s}+1}+\Delta t\psi u^{\tilde{s}}u_{5x_{k}}^{\tilde{s}+1}+\Delta tu_{7x_{k}}^{\tilde{s}+1}=u^{\tilde{s}}\\ & +3\alpha \Delta tu^{3\tilde{s}}u_{x_{k}}^{\tilde{s}}+2\beta \Delta tu_{x_{k}}^{3\tilde{s}}+2\gamma \Delta tu^{\tilde{s}}u_{x_{k}}^{\tilde{s}}u_{2x_{k}}^{\tilde{s}}+2\mu \Delta tu^{2\tilde{s}}u_{3x_{k}}^{\tilde{s}}+\nu \Delta tu_{2x_{k}}^{\tilde{s}}u_{3x_{k}}^{\tilde{s}}\\ & +\phi \Delta tu_{x_{k}}^{\tilde{s}}u_{4x_{k}}^{\tilde{s}}+\psi \Delta tu^{\tilde{s}}u_{5x_{k}}^{\tilde{s}}, \end{array} \end{array}$$ (20)

where $k=1,2,...,\tilde{S}$ are CPs.

The highest order partial derivative occurring in Eq (1) can be approximated using HW functions as:

$$\begin{array}{c} u_{7x}\left(x,t\right)=\sum_{\tilde{l}=1}^{\tilde{S}}{\overset{ˇ}{a}}_{\tilde{l}}\varsigma_{\tilde{l}}\left(x\right). \end{array}$$ (21)

Integration of Eq (21) w.r.t. *x* yields the interpretations for the other derivatives and the function i.e. *u*(*x*, *t*) as:

$$\begin{array}{c} u_{6x}\left(x,t\right)=u_{6x}\left(0,t\right)+\sum_{\tilde{l}=1}^{\tilde{S}}{\overset{ˇ}{a}}_{\tilde{l}}\delta_{\tilde{l},1}\left(x\right). \end{array}$$ (22)

$$\begin{array}{c} u_{5x}\left(x,t\right)=u_{5x}\left(0,t\right)+xu_{6x}\left(0,t\right)+\sum_{\tilde{l}=1}^{\tilde{S}}{\overset{ˇ}{a}}_{\tilde{l}}\delta_{\tilde{l},2}\left(x\right). \end{array}$$ (23)

$$\begin{array}{c} u_{4x}\left(x,t\right)=u_{4x}\left(0,t\right)+xu_{5x}\left(0,t\right)+\frac{x^{2}}{2}u_{6x}\left(0,t\right)+\sum_{\tilde{l}=1}^{\tilde{S}}{\overset{ˇ}{a}}_{\tilde{l}}\delta_{\tilde{l},3}\left(x\right). \end{array}$$ (24)

$$\begin{array}{c} \begin{array}{c} u_{3x}\left(x,t\right)=u_{3x}\left(0,t\right)+xu_{4x}\left(0,t\right)+\frac{x^{2}}{2}u_{5x}\left(0,t\right)+\frac{x^{3}}{6}u_{6x}\left(0,t\right)+\sum_{\tilde{l}=1}^{\tilde{S}}{\overset{ˇ}{a}}_{\tilde{l}}\delta_{\tilde{l},4}\left(x\right). \end{array} \end{array}$$ (25)

$$\begin{array}{c} \begin{array}{c} u_{2x}\left(x,t\right)=u_{2x}\left(0,t\right)+xu_{3x}\left(0,t\right)+\frac{x^{2}}{2}u_{4x}\left(0,t\right)+\frac{x^{3}}{6}u_{5x}\left(0,t\right)+\frac{x^{4}}{24}u_{6x}\left(0,t\right)+\sum_{\tilde{l}=1}^{\tilde{S}}{\overset{ˇ}{a}}_{\tilde{l}}\delta_{\tilde{l},5}\left(x\right). \end{array} \end{array}$$ (26)

$$\begin{array}{c} \begin{array}{cc} & u_{x}\left(x,t\right)=u_{x}\left(0,t\right)+xu_{2x}\left(0,t\right)+\frac{x^{2}}{2}u_{3x}\left(0,t\right)+\frac{x^{3}}{6}u_{4x}\left(0,t\right)+\frac{x^{4}}{24}u_{5x}\left(0,t\right)+\frac{x^{5}}{120}u_{6x}\left(0,t\right)\\ & +\sum_{\tilde{l}=1}^{\tilde{S}}{\overset{ˇ}{a}}_{\tilde{l}}\delta_{\tilde{l},6}\left(x\right). \end{array} \end{array}$$ (27)

$$\begin{array}{c} \begin{array}{cc} & u\left(x,t\right)=u\left(0,t\right)+xu_{x}\left(0,t\right)+\frac{x^{2}}{2}u_{2x}\left(0,t\right)+\frac{x^{3}}{6}u_{3x}\left(0,t\right)+\frac{x^{4}}{24}u_{4x}\left(0,t\right)+\frac{x^{5}}{120}u_{5x}\left(0,t\right)\\ & +\frac{x^{6}}{720}u_{6x}\left(0,t\right)+\sum_{\tilde{l}=1}^{\tilde{S}}{\overset{ˇ}{a}}_{\tilde{l}}\delta_{\tilde{l},7}\left(x\right). \end{array} \end{array}$$ (28)

The expressions for the unknowns *u*_4*x*_(0, *t*), *u*_5*x*_(0, *t*) and *u*_6*x*_(0, *t*) are calculated by integrating Eqs (25), (26) and (27) w.r.t. *x* from 0 to 1 as:

$$\begin{array}{c} \begin{array}{cc} & u_{4x}\left(0,t\right)=-360u\left(0,t\right)+360u\left(1,t\right)-240u_{x}\left(0,t\right)-120u_{x}\left(1,t\right)-72u_{2x}\left(0,t\right)\\ & +12u_{2x}\left(1,t\right)-12u_{3x}\left(0,t\right)-12\sum_{\tilde{l}=1}^{\tilde{S}}{\overset{ˇ}{a}}_{\tilde{l}}\delta_{\tilde{l},5}\left(1\right)+120\sum_{\tilde{l}=1}^{\tilde{S}}{\overset{ˇ}{a}}_{\tilde{l}}\delta_{\tilde{l},6}\left(1\right)-360\sum_{\tilde{l}=1}^{\tilde{S}}{\overset{ˇ}{a}}_{\tilde{l}}\delta_{\tilde{l},7}\left(1\right). \end{array} \end{array}$$ (29)

$$\begin{array}{c} \begin{array}{cc} & u_{5x}\left(0,t\right)=2880u\left(0,t\right)-2880u\left(1,t\right)+1800u_{x}\left(0,t\right)+1080u_{x}\left(1,t\right)+480u_{2x}\left(0,t\right)\\ & -120u_{2x}\left(1,t\right)+60u_{3x}\left(0,t\right)+120\sum_{\tilde{l}=1}^{\tilde{S}}{\overset{ˇ}{a}}_{\tilde{l}}\delta_{\tilde{l},5}\left(1\right)-1080\sum_{\tilde{l}=1}^{\tilde{S}}{\overset{ˇ}{a}}_{\tilde{l}}\delta_{\tilde{l},6}\left(1\right)+2880\sum_{\tilde{l}=1}^{\tilde{S}}{\overset{ˇ}{a}}_{\tilde{l}}\delta_{\tilde{l},7}\left(1\right). \end{array} \end{array}$$ (30)

$$\begin{array}{c} \begin{array}{cc} & u_{6x}\left(0,t\right)=-7200u\left(0,t\right)+7200u\left(1,t\right)-4320u_{x}\left(0,t\right)-2880u_{x}\left(1,t\right)-1080u_{2x}\left(0,t\right)\\ & +360u_{2x}\left(1,t\right)-120u_{3x}\left(0,t\right)-360\sum_{\tilde{l}=1}^{\tilde{S}}{\overset{ˇ}{a}}_{\tilde{l}}\delta_{\tilde{l},5}\left(1\right)+2880\sum_{\tilde{l}=1}^{\tilde{S}}{\overset{ˇ}{a}}_{\tilde{l}}\delta_{\tilde{l},6}\left(1\right)-7200\sum_{\tilde{l}=1}^{\tilde{S}}{\overset{ˇ}{a}}_{\tilde{l}}\delta_{\tilde{l},7}\left(1\right). \end{array} \end{array}$$ (31)

Substituting all the above expressions in Eq (20), we obtain a system of equations. In this system of equations, the known values of *u*_*k*_’s at time $t_{\tilde{s}}$ are substituted to calculate the Haar coefficients for the solution at the next time $t_{\tilde{s}+1}$. Actual solution at the next time is obtained using these Haar coefficients. Using this iterative process, the solution at any specific time can be obtained.

## 4 Convergence theorem

**Theorem 1** *Suppose that* $f\left(x\right)=\frac{d^{\tilde{s}}u\left(x\right)}{dx^{\tilde{s}}}\in L^{2}\left(R\right)$ *is a continuous function on* [0, 1] *and its first derivative is bounded*:

$$\begin{array}{c} \forall \;x\in \left[0,1\right],\;\exists \;\varrho :|\frac{df(x)}{d\rho }|\leq \varrho ,\;\tilde{s}\geq 2. \end{array}$$

*Then the HW method, based on approach proposed in* [30, 49] *will be convergent, i.e*., $|E_{\tilde{r}}|$ *vanishes as* $\tilde{M}$ *tends to infinity, the convergence is of order two*:

$$\begin{array}{c} ∥E_{\tilde{r}}{∥}_{2}=O[{\left(\frac{1}{2^{\tilde{M}+1}}\right)}^{2}], \end{array}$$

*where*

$$\begin{array}{c} E_{\tilde{r}}=f\left(x\right)-f_{\tilde{R}}\left(x\right),\;f_{\tilde{R}}\left(x\right)=\sum_{\tilde{l}=0}^{\tilde{S}}{\overset{ˇ}{a}}_{\tilde{l}}\varsigma_{\tilde{l}}\left(x\right). \end{array}$$

Proof: For proof, see [30, 50].

## 5 Results and discussion

To analyze the validity of the presented numerical technique, some test problems are considered in this section. The maximum absolute errors (MAEs) are denoted by the symbol $E_{c}\left(\tilde{S}\right)$. Here, all the problems are defined on the interval [0, 1].

**Test Problem 1** *Consider the Lax equation of order seven which is given in* Eq (4) with exact solution [1, 2, 45]:

$$\begin{array}{c} u\left(x,t\right)=2{\tilde{j}}^{2}sech^{2}(\tilde{j}x-64{\tilde{j}}^{7}t), \end{array}$$ (32)

*where x is space variable and t is temporal variable*, *u*(*x*, *t*) *is an unknown function*, $\tilde{j}$ *is an arbitrary parameter*, Ψ *is the domain and* ∂Ψ *is the boundary. The IC and BCs are obtained from the exact solution. The seventh-order Lax equation is a nonlinear heat equation that has hyperbolic function as its exact solution. In the physical phenomena the change of long 1-D waves is determined by the prescribed heat equation that includes shallow water waves. Similarly, in our daily life, the physical part of hyperbolic functions is significant, as they can be used to describe the shape of the curves formed by a high-voltage line suspended between two towers. The* Tables 1 and 2 *represent the computed results obtained by applying 1-D HWCM on the* Eq (4).

**Table 1:** Point wise errors at Δ*t* = 0.01 and $\tilde{j}=0.3$ and $\tilde{S}=32$ for Test Problem 1.

*x*/*t*	2	4	6	8	10
	Point wise error	Point wise error	Point wise error	Point wise error	Point wise error
0	0	0	0	0	0
0.1	2.0853 × 10^−07^	8.9657 × 10^−07^	2.0534 × 10^−06^	3.6617 × 10^−06^	5.6972 × 10^−06^
0.2	4.2940 × 10^−07^	2.5373 × 10^−06^	6.2873 × 10^−06^	1.1624 × 10^−05^	1.8469 × 10^−05^
0.3	6.7499 × 10^−08^	2.9241 × 10^−06^	8.9113 × 10^−06^	1.7807 × 10^−05^	2.9482 × 10^−05^
0.4	1.6779 × 10^−06^	7.0797 × 10^−07^	7.0813 × 10^−06^	1.7351 × 10^−05^	3.1368 × 10^−05^
0.5	4.1240 × 10^−06^	3.9311 × 10^−06^	5.1438 × 10^−07^	9.1502 × 10^−06^	2.1853 × 10^−05^
0.6	6.4536 × 10^−06^	9.1709 × 10^−06^	8.1807 × 10^−06^	3.4949 × 10^−06^	4.8213 × 10^−06^
0.7	7.3758 × 10^−06^	1.2199 × 10^−05^	1.4456 × 10^−05^	1.4111 × 10^−05^	1.1167 × 10^−05^
0.8	5.9278 × 10^−06^	1.0626 × 10^−05^	1.4055 × 10^−05^	1.6165 × 10^−05^	1.6924 × 10^−05^
0.9	2.4696 × 10^−06^	4.6532 × 10^−06^	6.5259 × 10^−06^	8.0602 × 10^−06^	9.2334 × 10^−06^
1	3.8775 × 10^−14^	7.5662 × 10^−14^	1.0966 × 10^−13^	1.4139 × 10^−13^	1.6878 × 10^−13^

**Table 2:** Maximum absolute errors at *t* = 1 and $\tilde{j}=0.3$ for Test Problem 1.

$\tilde{S}$	Δ*t* = 0.1	Δ*t* = 0.01	Δ*t* = 0.001
	*L*_∞_	*L*_∞_	*L*_∞_
2	3.7267 × 10^−06^	3.7267 × 10^−06^	3.7266 × 10^−06^
4	3.8872 × 10^−06^	3.8874 × 10^−06^	3.8874 × 10^−06^
8	4.0451 × 10^−06^	4.0452 × 10^−06^	4.0452 × 10^−06^
16	4.0210 × 10^−06^	4.0211 × 10^−06^	4.0211 × 10^−06^
32	4.0476 × 10^−06^	4.0477 × 10^−06^	4.0477 × 10^−06^
64	4.0502 × 10^−06^	4.0503 × 10^−06^	4.0503 × 10^−06^
128	4.0498 × 10^−06^	4.0499 × 10^−06^	4.0499 × 10^−06^

**Fig 1:**
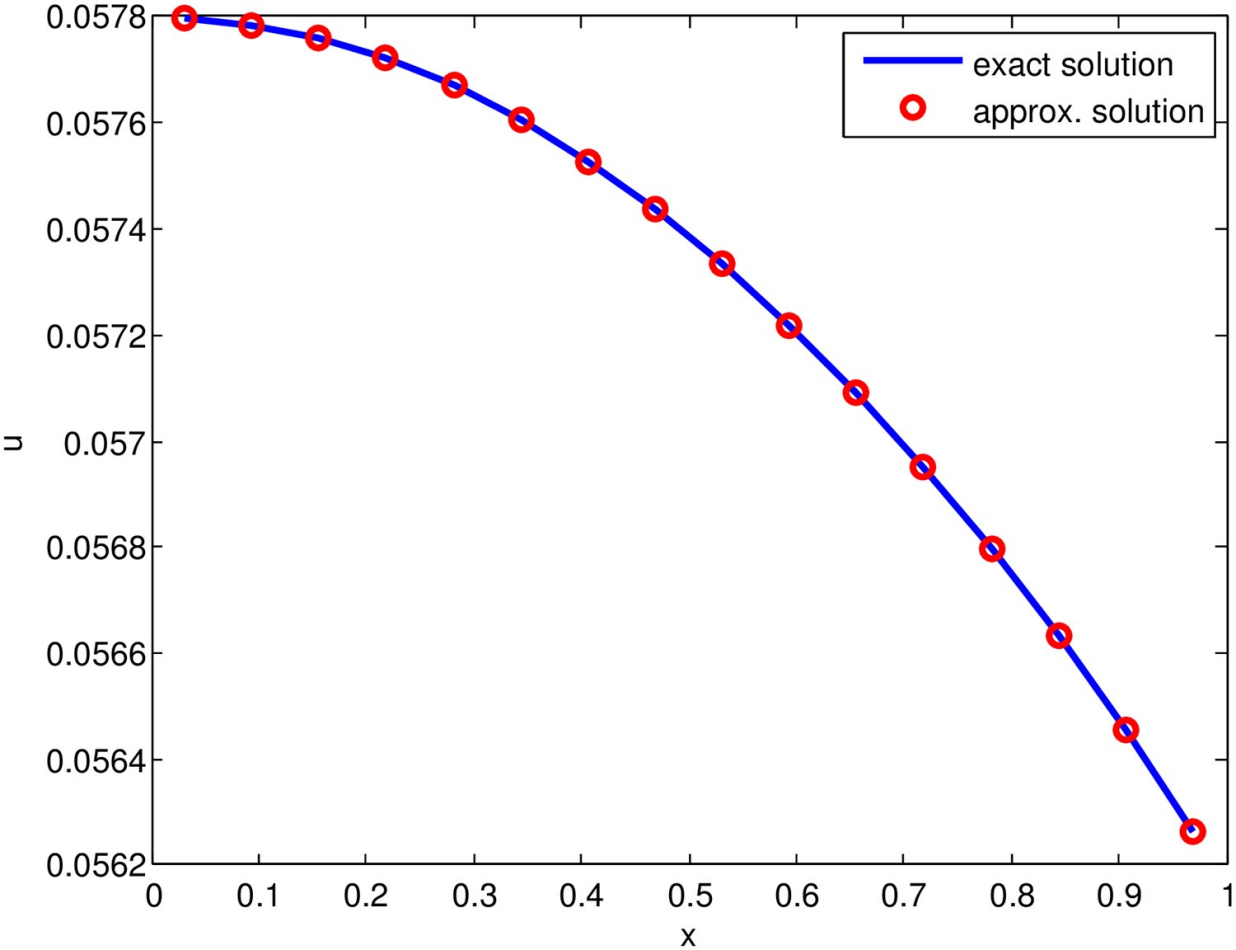
Comparison of approximate solution (for $\tilde{S}=16$, *t* = 1, Δ*t* = 0.01 and $\tilde{j}=0.17$) and exact solution for Test Problem 1.

*The* Table 1 *indicates the point wise absolute errors for different time levels. It is observed that at x* = 0 and *x* = 1 *the absolute errors are zero, whereas, the other errors are increased with the increase in time levels (by passage of time),that assures the property of time marching scheme*. *In* Table 2, *for different values of* Δ*t* (*time step*) *and the time level t* = 1, *the MAEs at CPs have been calculated. The accuracy of the proposed method is measured by using different CPs, that guarantees the computational cost of 1-D HWCM is small. The point wise absolute errors are diminished up to the order* 10^−13^ *with* Δ*t* = 0.01 *and x* = 0.3. *The MAEs are diminished up to the order* 10^−06^ *and increased with the increasing time, that confirms the adequateness of the time marching scheme. It is noticeable, that after reducing the time step the accuracy of the presented scheme is increased. Moreover, the exact solution and approximate solution are shown graphically in* Fig 1.

**Test Problem 2** *Consider the Sawada-Kotera-Ito equation of order seven given in* Eq (5) *with exact solution* [51]:

$$\begin{array}{c} u\left(x,t\right)=\frac{4{\tilde{j}}^{2}}{3}(2-3tanh^{2}(\tilde{j}x+\frac{256}{3}{\tilde{j}}^{7}t)), \end{array}$$ (33)

*where x is space variable and t is temporal variable*, *u*(*x*, *t*) *is an unknown function*, $\tilde{j}$ *is an arbitrary parameter*, Ψ *is the domain and* ∂Ψ *is the boundary. The IC and BCs are obtained from the exact solution. The Sawada-Kotera-Ito equation is a nonlinear parabolic PDE with tangent hyperbolic function as its exact solution. By applying 1-D HWCM, the computational outcomes are illustrated in* Tables 3 and 4.

**Table 3:** Point wise errors at Δ*t* = 0.01, $\tilde{j}=0.4$ and $\tilde{S}=32$ for Test Problem 2.

*x*/*t*	1	5	10
	Point wise error	Point wise error	Point wise error
0	0	0	0
0.1	5.4655 × 10^−09^	1.0357 × 10^−08^	4.3205 × 10^−09^
0.2	5.1077 × 10^−08^	1.1329 × 10^−07^	4.9431 × 10^−08^
0.3	1.3776 × 10^−07^	3.7377 × 10^−07^	1.7068 × 10^−07^
0.4	2.0330 × 10^−06^	7.2330 × 10^−07^	3.4586 × 10^−07^
0.5	1.8730 × 10^−06^	9.9333 × 10^−07^	4.9760 × 10^−07^
0.6	9.3628 × 10^−07^	1.0250 × 10^−06^	5.8108 × 10^−07^
0.7	7.9367 × 10^−09^	7.7874 × 10^−07^	4.2852 × 10^−07^
0.8	4.4239 × 10^−08^	3.8274 × 10^−07^	2.2078 × 10^−07^
0.9	1.6723 × 10^−08^	7.4554 × 10^−08^	4.5079 × 10^−08^
1	7.7716 × 10^−16^	1.3878 × 10^−16^	3.0531 × 10^−16^

**Table 4:** Maximum absolute errors at *t* = 1 and $\tilde{j}=0.4$ for Test Problem 2.

$\tilde{S}$	Δ*t* = 0.1	Δ*t* = 0.01	Δ*t* = 0.001
	*L*_∞_	*L*_∞_	*L*_∞_
2	1.0848 × 10^−06^	7.0137 × 10^−07^	6.5995 × 10^−07^
4	1.9637 × 10^−06^	5.3915 × 10^−06^	3.7997 × 10^−07^
8	2.1400 × 10^−06^	3.2034 × 10^−07^	1.6643 × 10^−07^
16	2.1656 × 10^−06^	2.2942 × 10^−07^	1.7598 × 10^−07^
32	2.1873 × 10^−06^	2.0788 × 10^−07^	2.0465 × 10^−07^
64	2.1901 × 10^−06^	2.0245 × 10^−07^	2.1203 × 10^−07^
128	2.1910 × 10^−06^	2.0088 × 10^−07^	2.1403 × 10^−07^

*In* Table 3, *the point wise absolute errors have been calculated at the values of x* = 0 *to* 1 *and t* = 1, 5, 10 *for x* = 0 *and x* = 1, *it is observed that its too near to the exact solution as the absolute errors (AEs) at these two points are zero and for the other points in the interval* [0, 1], *the AEs are increased gradually. Moreover, in* Table 4, *MAEs are calculated using different CPs taking different values of time step i.e*. Δ*t* = 0.1, 0.01 *and* 0.001, $\tilde{n}=0.4$ *and t* = 1.

*The MAEs are decreased upto* 10^−06^ *for different CPs with different time steps* Δ*t* = 0.1, 0.01 and 0.001. *Moreover, the adequate behavior of the prescribed scheme may be monitored by less computational cost. It can also be noticed from the* Tables 3 and 4, *that the point wise errors are increased by increasing values of x and MAEs are reduced by reducing* Δ*t*.

**Fig 2:**
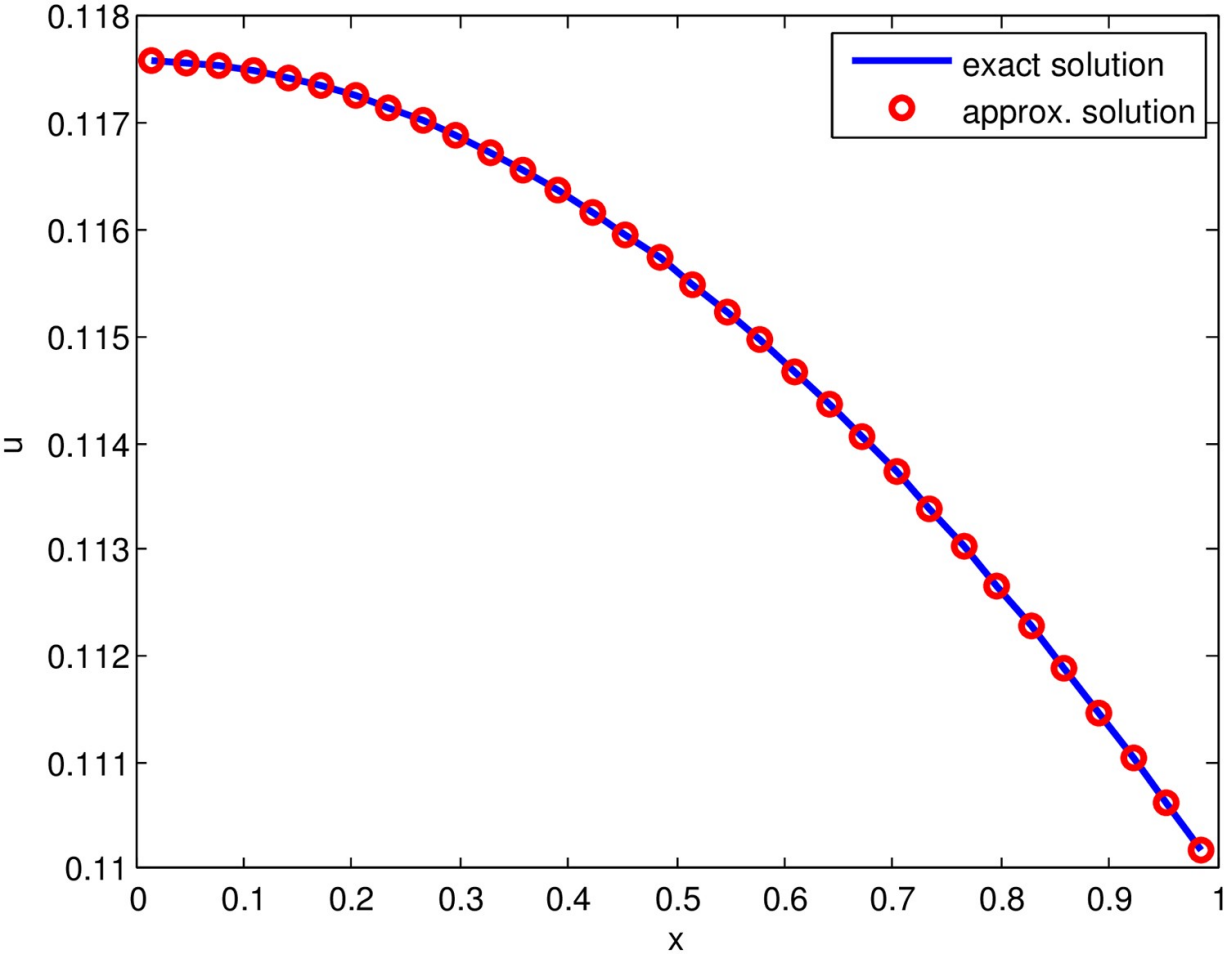
Comparison of approximate solution (for $\tilde{S}=32$, *t* = 1, Δ*t* = 0.01 and $\tilde{j}=0.21$) and exact solution for Test Problem 2.

*The* Fig 2, *illustrates the graphical behavior of approximate solution (for* $\tilde{S}=32$) *and exact solution*.

**Test Problem 3** *Consider the Kaup-Kuperschmidt equation of order seven given in* Eq (6) *with exact solution* [1]:

$$\begin{array}{c} u\left(x,t\right)=\frac{1}{3}{\tilde{j}}^{2}-\frac{1}{2}{\tilde{j}}^{2}tanh^{2}(\tilde{j}x+\frac{4}{3}{\tilde{j}}^{7}t), \end{array}$$ (34)

*where x is space variable and t is temporal variable*, *u*(*x*, *t*) *is an unknown function*, $\tilde{j}$ *is an arbitrary parameter*, Ψ *is the domain and* ∂Ψ *is the boundary. The IC and BCs are obtained from the exact solution. The Kaup-Kuperschmidt equation is a nonlinear heat equation that plays a significant role in mathematical physics, optical fibers, solid state physics, fluid dynamics and so on. The exact solution of this equation is also tangent hyperbolic function that has its significant effects in our daily life*.

**Fig 3:**
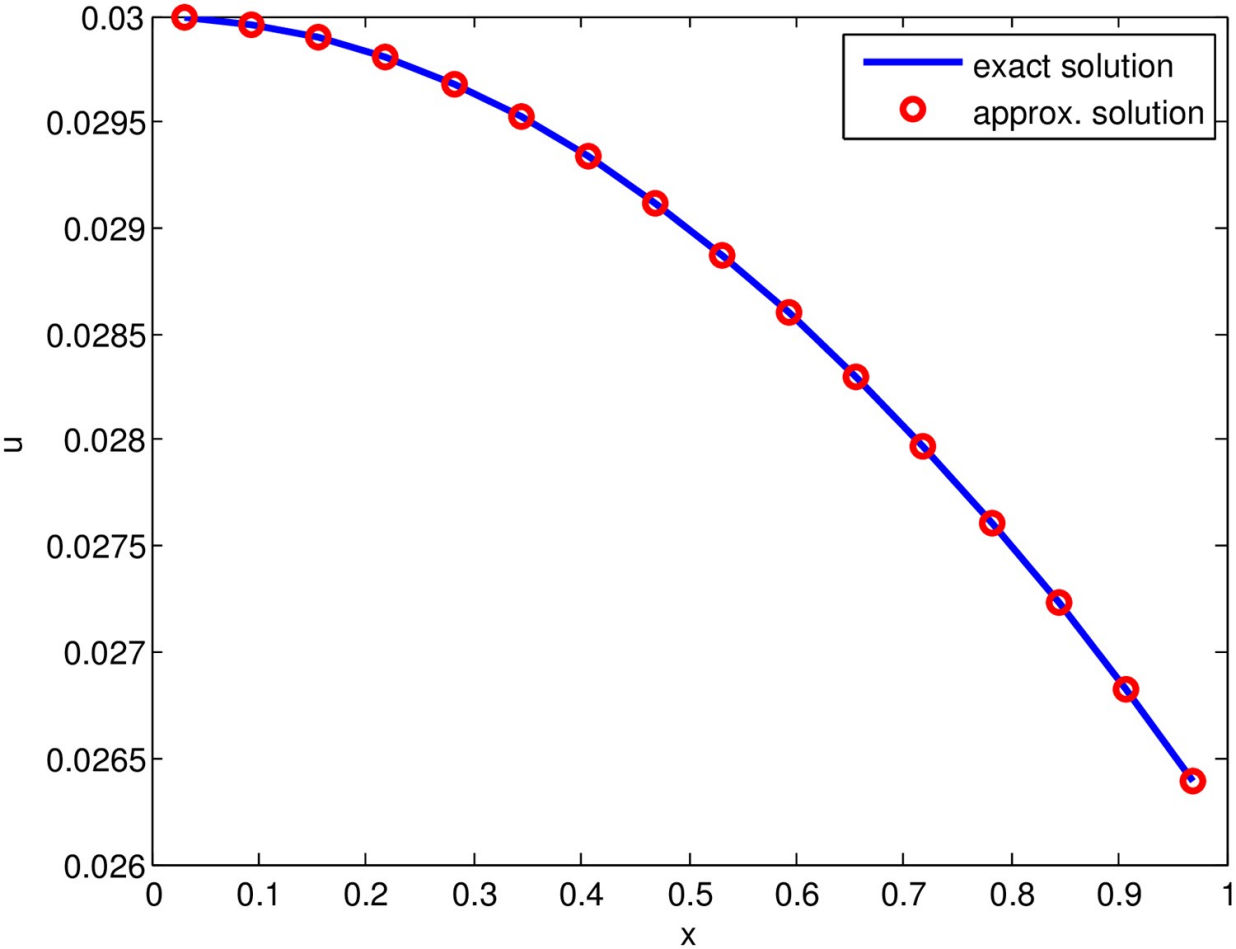
Comparison of approximate solution (for $\tilde{S}=32$, *t* = 1, Δ*t* = 0.01 and $\tilde{j}=0.21$) and exact solution for Test Problem 3.

*The* Table 5 *shows the computed results of this problem. The MAEs are illustrated in* Table 5, *for different values of time step* Δ*t* = 0.1, 0.01 and 0.001. *The MAEs are diminished upto the order* 10^−06^. *Moreover, the* Table 5 *indicates that the property of time marching scheme is satisfied in this test problem also. The approximate solution and exact solution are presented graphically in* Fig 3, *that shows the vigorous behavior of the proposed scheme*.

**Table 5:** Maximum absolute errors at *t* = 1 and $\tilde{j}=0.3$ for Test Problem 3.

$\tilde{S}$	Δ*t* = 0.1	Δ*t* = 0.01	Δ*t* = 0.001
	*L*_∞_	*L*_∞_	*L*_∞_
2	1.4494 × 10^−06^	1.1149 × 10^−06^	1.0818 × 10^−06^
4	2.9750 × 10^−06^	1.9555 × 10^−06^	1.8531 × 10^−06^
8	3.2199 × 10^−06^	2.0742 × 10^−06^	1.9591 × 10^−06^
16	3.2428 × 10^−06^	2.0736 × 10^−06^	1.9562 × 10^−06^
32	3.2689 × 10^−06^	2.0893 × 10^−06^	1.9708 × 10^−06^
64	3.2710 × 10^−06^	2.0909 × 10^−06^	1.9723 × 10^−06^
128	3.2723 × 10^−06^	2.0911 × 10^−06^	1.9724 × 10^−06^

## 6 Conclusion

Some nonlinear PDEs of order seven are approximated including one-dimensional Lax, Sawada-Kotera-Ito and Kaup-Kuperschmidt equations. The exact solution is approximated by the one-dimensional HWCM. The proposed general equation is discretized using FDM and the collocation procedure. The proposed scheme is applied upon different test problems and an economical conduct of the scheme is investigated from the computed results. In the test problems, by reducing the time step, the accuracy is increased.

## Supporting information

S1 File (DOCX)

## References

[1] Arora R, Sharma H. Application of HAM to seventh order KdV equations. Int. J. Syst. Assur. Eng. Manag. 2018;9(1):131–138. 10.1007/s13198-016-0490-7

[2] Darvishi MT, Kheybari S, Khani F. A Numerical Solution of the Lax’s 7th-order KdV equation by Pseudospectral Method and Darvishi’s Preconditioning. Int. J. Contemp. Math. Sci. 2007;2(22):1097–1106.

[3] Wu X, Tian SF, Yang JJ. Riemann-Hilbert Approach and N-Soliton Solutions For Three-Component Coupled Hirota Equations. East Asian J. Appl. Math. 2020;10(4):717–731. 10.4208/eajam.170120.080420

[4] Peng WQ, Tian SF, Zhang TT. Initial Value Problem for the Pair Transition Coupled Nonlinear Schrödinger Equations via the Riemann-Hilbert Method. Complex. Anal. Operat. Theo. 2020;14:38 10.1007/s11785-020-00997-1

[5] Mao JJ, Tian SF, Zhang TT, Yan XJ. Lie symmetry analysis, conservation laws and analytical solutions for chiral nonlinear Schrödinger equation in (2+1)-dimensions. Nonlin. Anal: Model. Cont. 2020;25(3):358–377.

[6] Tian SF, Yang JJ, Li ZQ, Chen YR. Blow-up phenomena of a weakly dissipative modified two-component DullinGottwaldHolm system. Appl. Math. Lett. 2020;106:106378 10.1016/j.aml.2020.106378

[7] Tian SF. Lie symmetry analysis, conservation laws and solitary wave solutions to a fourth-order nonlinear generalized Boussinesq water wave equation. Appl. Math. Lett. 2020;100:106056 10.1016/j.aml.2019.106056

[8] Xu TY, Tian SF, Peng WQ. Riemann-Hilbert approach for multisoliton solutions of generalized coupled fourth-order nonlinear Schrödinger equations. Math. Meth. Appl. Sci. 2020;43(2):865–880. 10.1002/mma.5964

[9] Peng WQ, Tian SF, Zhang TT. Dynamics of the soliton waves, breather waves, and rogue waves to the cylindrical Kadomtsev-Petviashvili equation in pair-ion electron plasma. Phys. Fluids 2019;31(10):102107 10.1063/1.5116231

[10] Peng WQ, Tian SF, Wang XB, Zhang TT, Fang Y. RiemannHilbert method and multi-soliton solutions for three-component coupled nonlinear Schrödinger equations. J. Geom. Phys. 2019;146:103508 10.1016/j.geomphys.2019.103508

[11] Pomeau Y, Ramani A, Grammaticos B. Structural stability of the Korteweg-de Vries solitons under a singular perturbation. Physica D. 1988;31(1):127–134. 10.1016/0167-2789(88)90018-8

[12] Fermo L, Mee C, Seatzu S. A numerical method to compute the scattering solution for the KdV equation. Appl. Nume. Math. 2020;149:3–16. 10.1016/j.apnum.2019.07.001

[13] Kong D, Xu Y, Zheng Z. A hybrid numerical method for the KdV equation by finite difference and sinc collocation method. Appl. Math. Comput. 2019;355:61–72.

[14] Saravi M, Nikkar A, Hermann M, Vahidi J, Ahari R. A New Modified Approach for solving seven-order Sawada-Kotara equations. J. Math. Comp. Sci. 2013;6:230–237. 10.22436/jmcs.06.03.07

[15] Zhang LD, Tian SF, Peng WQ, Zhang TT, Yan XJ. The Dynamics of Lump, Lumpoff and Rogue Wave Solutions of (2+1)-Dimensional Hirota-Satsuma-Ito Equations. East Asian J. Appl. Math. 2020;10(2):243–255. 10.4208/eajam.130219.290819

[16] Feng LL, Tian SF, Zhang TT. Bäckland Transformations, Nonlocal Symmetries and Soliton-Cnoidal Interaction Solutions of the (2+1)-Dimensional Boussinesq Equation. Bull. Malays. Math. Sci. Soc. 2020;43(1):141–155. 10.1007/s40840-018-0668-z

[17] Peng WQ, Tian SF, Zhang TT, Fang Y. Rational and semi-rational solutions of a nonlocal (2+1)-dimennsional nonlinear Schrödinger equation. Math. Meth. Appl. Sci. 2019;42(18):6865–6877. 10.1002/mma.5792

[18] Aljahdaly NH, Seadawy AR, Albarakati WA. Applications of dispersive analytical wave solutions of nonlinear seventh order Lax and Kaup-Kuperschmidt dynamical wave equations. Results in Phy. 2019;14:102372 10.1016/j.rinp.2019.102372

[19] Ganji DD, Abdollahzadeh M. Exact travelling solutions for the Lax’s seventh-order KdV equation by sech method and rational exp-function method. Appl. Math. Comput. 2008;206:438–444.

[20] Sharma A, Arora R. Solutions of Fisher-Type, Cubic-Boussinesq and 7th-Order Caudrey-Dodd-Gibbon Equations by MVIM. Int. J. Appl. Comput. Math. 2017;3:3857–3875. 10.1007/s40819-017-0332-6

[21] Salas AH. Computing exact solutions to a generalized Lax seventh-order forced KdV equation (KdV7). Appl. Math. Comput. 2010;216:2333–2338.

[22] Cattani C, Kudreyko A. Harmonic wavelet method towards solution of the Fredholm type integral equations of the second kind. Appl. Math. Comput. 2010;215:4164–4171.

[23] Cattani C. A review on Harmonic wavelets and their fractional extension. J. Adv. Eng. Comput. 2018;2(4):224–238. 10.25073/jaec.201824.225

[24] Grossmann A, Morlet J. Decomposition of Hardy Functions into Square Integrable Wavelets of Constant Shape. SIAM J. Math. Anal. 1984;15:723–736. 10.1137/0515056

[25] Meyer Y. Analysis at Urbana: Volume 1, Analysis in Function Spaces. Camb. Uni. Press, ISBN: 978-0-521-36436-2, 1989.

[26] Mallat SG. Multiresolution approximations and wavelet orthonormal bases of *L*_2_(*R*), Trans. Amer. Math. Soc. 1989;315(1):69–87. 10.1090/S0002-9947-1989-1008470-5

[27] Siraj-ul-Islam, Aziz I, Šarler B. The numerical solution of second-order boundary-value problems by collocation method with the Haar wavelets. Math. Comput. Model. 2010;52:1577–1590. 10.1016/j.mcm.2010.06.023

[28] Haar A. Zur theorie der orthogonalen funktionen systeme. Math. Ann. 1910;69:331–371. 10.1007/BF01456326

[29] Aziz I, Siraj-ul-Islam, Šarler B. Wavelets collocation methods for the numerical solution of elliptic BV problems. Appl. Math. Model. 2013;37:676–694. 10.1016/j.apm.2012.02.046

[30] Majak J, Shvartsman B, Kirs M, Pohlak M, Herranen H. Convergence theorem for the Haar wavelet based discretization method. Compos. Struct. 2015;126(1):227–232.

[31] Lepik Ü. Numerical solution of differential equations using Haar wavelets. Math. Comput. Simul. 2005;68(2):127–143. 10.1016/j.matcom.2004.10.005

[32] Lepik Ü. Haar wavelet method for nonlinear integro-differential equations. Appl. Math. Comput. 2006;176(1):324–333.

[33] Majak J, Pohlak M, Eerme E, Lepikult T. Weak formulation based Haar wavelet method for solving differential equations. Appl. Math. Comput. 2009;211(2):488–494.

[34] Majak J, Pohlak M, Karjust K, Eerme M, Kurnistki J, Shvartsman B. New higher order Haar wavelet method: Application to FGM structures. Compos. Struct. 2018;201:72–78. 10.1016/j.compstruct.2018.06.013

[35] Mohanty RK. A fourth-order finite difference method for the general one-dimensional nonlinear biharmonic problems of first kind. J. Comput. Appl. Math. 2000;114(2):275–290. 10.1016/S0377-0427(99)00202-2

[36] Mohanty RK, Jain MK. Technical note: The numerical solution of the system of 3-D nonlinear elliptic equations with mixed derivatives and variable coefficients using fourth-order difference methods. Numer. Meth. Partial Diff. Eqs. 1995;11:187–197. 10.1002/num.1690110303

[37] Mohanty RK, Singh S. High accuracy numerov type discretization for the solution of one-space dimensional nonlinear wave equations with variable coefficients. J. Adv. Res. Sci. Comput. 2011;3(1):53–66.

[38] Yokuş A. Comparison of caputo and conformable derivatives for time-fractional Kortweg-de Vries equation via finite difference method. Int. J. Mod. Phys B 2018;32(29):1–12.

[39] Yokuş A. Numerical solution for space and time fractional order Burger type equation. Alex. Eng. J. 2018;57(3):2085–2091. 10.1016/j.aej.2017.05.028

[40] Kaya D, Gulbahar S, Yokuş A, Gulbahar M. Solutions of the fractional combined KdV-mKdV equation with collocation method using radial basis function and their geometrical obstructions. Adv. Differ. Equ. 2018;77:1–16.

[41] Yokuş A. Numerical solutions of Time Fractional Korteweg de Vries equation and its Stability Analysis. Commun. Facul. Sci. Uni. Ankara Ser. A1 Math. Stat. 2019;68(1):353–361.

[42] Kaya D, Gulbahar S, Yokuş A. Numerical solutions of the fractional KdV-Burgers-Kuramoto equation. Ther. Sci. 2018;22(1):S153–S158.

[43] Aziz I, Siraj-ul-Islam, Khan W. Quadrature rules for numerical integration based on Haar wavelets and hybrid functions. Comput. Math. Appl. 2011;61:2770–2781. 10.1016/j.camwa.2011.03.043

[44] Lepik Ü. Numerical solution of evolution equations by the Haar wavelet method. Appl. Math. Comput. 2007;185(1):695–704.

[45] El-Sayed SM, Kaya D. An application of the ADM to seven-order SawadaKotara equations. Appl. Math. Comput. 2004;157:93–101.

[46] Wazwaz AM. Exact travelling wave solutions to seventh-order and ninth-order KdV-like equations. Appl. Math. Comput. 2006;182:771–780.

[47] Saleem S, Numerical Solution of Time-Dependent Partial Differential Equations Via Haar Wavelet, Ph.D. Thesis, Department of Mathematics, University of the Punjab, Lahore, Pakistan 2020;1–122.

[48] Chen CF, Hsiao CH. Haar wavelet method for solving lumped and distributed-parameter systems. IEE Proc. Control Theo. Appl. 1997;144(1):87–94. 10.1049/ip-cta:19970702

[49] Hsiao CH. State analysis of the linear time delayed systems via Haar wavelets. Math. Comput. Simul. 1997;44(5):457–470. 10.1016/S0378-4754(97)00075-X

[50] Majak J, Shvartsman B, Karjust K, Mikola M, Haavajoe A, Pohlak M. On the accuracy of the Haar wavelet discretization method. Compos. Part B. 2015;80:321–327. 10.1016/j.compositesb.2015.06.008

[51] Akinyemi L. q-Homotopy analysis method for solving the seventh-order time-fractional Lax’s Korteweg-de Vries and SawadaKotera equations. Comput. Appl. Math. 2019;38:191 10.1007/s40314-019-0977-3

