# Supplementary material for: A reliable algorithm to compute the approximate solution of KdV-type partial differential equations of order seven
Source: PLoS One. 2021 Jan 22;16(1):e0244027. doi: 10.1371/journal.pone.0244027 (PMC7822283; doi:10.1371/journal.pone.0244027)
Supplement: S1 File — (DOCX) [file pone.0244027.s001.docx]

**Additional Requirements of PLOS ONE**

**Journal:** PLOS ONE

**Paper Title:** A RELIABLE ALGORITHM TO COMPUTE THE APPROXIMATE SOLUTION OF KDV-TYPE PARTIAL DIFFERENTIAL EQUATIONS OF ORDER SEVEN

**Authors:** Sidra Saleem, Malik Zawwar Hussain, Imran Aziz

**Manuscript ID:** PONE-D-20-26537

| **Additional Requirements** | | |
| --- | --- | --- |
| 1. | Please ensure that your manuscript meets PLOS ONE's style requirements, including those for file naming. The PLOS ONE style templates can be found at  https://journals.plos.org/plosone/s/file?id=wjVg/PLOSOne_formatting_sample_main_body.pdf and  https://journals.plos.org/plosone/s/file?id=ba62/PLOSOne_formatting_sample_title_authors_affiliations.pdf | Since the manuscript is written in the PLOS ONE style template, it meets the requirements of PLOS ONE’s style. |
| 2. | a. We suggest you thoroughly copyedit your manuscript for language usage, spelling, and grammar. If you do not know anyone who can help you do this, you may wish to consider employing a professional scientific editing service.    Whilst you may use any professional scientific editing service of your choice, PLOS has partnered with both American Journal Experts (AJE) and Editage to provide discounted services to PLOS authors. Both organizations have experience helping authors meet PLOS guidelines and can provide language editing, translation, manuscript formatting, and figure formatting to ensure your manuscript meets our submission guidelines. To take advantage of our partnership with AJE, visit the AJE website (http://learn.aje.com/plos/) for a 15% discount off AJE services. To take advantage of our partnership with Editage, visit the Editage website (www.editage.com) and enter referral code PLOSEDIT for a 15% discount off Editage services.  If the PLOS editorial team finds any language issues in text that either AJE or Editage has edited, the service provider will re-edit the text for free.  b. Upon resubmission, please provide the following:   - The name of the colleague or the details of the professional service that edited your manuscript - A copy of your manuscript showing your changes by either highlighting them or using track changes (uploaded as a *supporting information* file) - A clean copy of the edited manuscript (uploaded as the new *manuscript* file) | 1. The co-authors have a good knowledge of English language (that is, for language usage, spelling and grammar). 2. Upon resubmission:  - The author along co-authors edited the manuscript. - A copy of manuscript showing changes by either highlighting them or using track changes will be uploaded as ‘supporting information’ file. - A clean copy of edited manuscript will be uploaded as ‘manuscript’ file |
| 3. | Thank you for stating the following financial disclosure:   [N/A].  At this time, please address the following queries:   1. Please clarify the sources of funding (financial or material support) for your study. List the grants or organizations that supported your study, including funding received from your institution. 2. State what role the funders took in the study. If the funders had no role in your study, please state: “The funders had no role in study design, data collection and analysis, decision to publish, or preparation of the manuscript.” 3. If any authors received a salary from any of your funders, please state which authors and which funders. 4. If you did not receive any funding for this study, please state: “The authors received no specific funding for this work.”  - Please include your amended statements within your cover letter; we will change the online submission form on your behalf. | The following queries are addressed as follows:   1. It is stated that there is no source of funding (financial or material support) for my study. Since, my study is not funded by any organization, so, there exists no list including my institution. 2. As my study is not funded by any organization, so, “the funders had no role in study design, data collection and analysis, decision to publish, or preparation of the manuscript”. 3. I have not received any funding. 4. Yes, the author did not receive any funding for this study and it is stated that, “The authors received no specific funding for this work”.  - Okay these amended statements will be included in the cover letter. |
| 4. | In your Data Availability statement, you have not specified where the minimal data set underlying the results described in your manuscript can be found. PLOS defines a study's minimal data set as the underlying data used to reach the conclusions drawn in the manuscript and any additional data required to replicate the reported study findings in their entirety. All PLOS journals require that the minimal data set be made fully available. For more information about our data policy, please see http://journals.plos.org/plosone/s/data-availability.  Upon re-submitting your revised manuscript, please upload your study’s minimal underlying data set as either Supporting Information files or to a stable, public repository and include the relevant URLs, DOIs, or accession numbers within your revised cover letter. For a list of acceptable repositories, please see http://journals.plos.org/plosone/s/data-availability#loc-recommended-repositories. Any potentially identifying patient information must be fully anonymized.  Important: If there are ethical or legal restrictions to sharing your data publicly, please explain these restrictions in detail. Please see our guidelines for more information on what we consider unacceptable restrictions to publicly sharing data: http://journals.plos.org/plosone/s/data-availability#loc-unacceptable-data-access-restrictions. Note that it is not acceptable for the authors to be the sole named individuals responsible for ensuring data access.  We will update your Data Availability statement to reflect the information you provide in your cover letter.   - Important: If there are ethical or legal restrictions to sharing your data publicly, please explain these restrictions in detail. Please see our guidelines for more information on what we consider unacceptable restrictions to publicly sharing data: <http://journals.plos.org/plosone/s/data-availability#loc-unacceptable-data-access-restrictions>. - Note that it is not acceptable for the authors to be the sole named individuals responsible for ensuring data access. | The minimal data set underlying the results described in our manuscript are found by two soft wares, MATLAB and MAPLE Package. MATLAB is used for calculations of point wise errors and maximum absolute errors and MAPLE Package is used for confirmation of exact solutions and finding derivatives for the calculating the boundary conditions.  We have taken the data (in the form of exact solutions of seventh order KdV-type differential equations, theorem and definitions) from the literature, given by the following references with URLs or DOIs:   - Aljahdaly NH, Seadawy AR, Albarakati WA. Applications of dispersive   analytical wave solutions of nonlinear seventh order Lax and Kaup-  Kuperschmidt  dynamical wave equations. Results  in Phy. 2019;14:102372.  It has URL as follows: <https://doi.org/10.1016/j.rinp.2019.102372>     - El-Sayed SM, Kaya D. An application of the ADM to seven-order Sawada Kotara   equations. Appl. Math. Comput.  2004;157:93-101.  It has DOI as follows:  doi:10.1016/j.amc.2003.08.104   - Majak J, Shvartsman B, Kirs M, Pohlak M, Herranen H. Convergence theorem   for the Haar wavelet based discretization method.Compos. Struct.2015;126(1):227-232.  It has URL as follows:  <http://dx.doi.org/10.1016/j.compstruct.2015.02.050>   - Arora R, Sharma H. Application of HAM to seventh order KdV equations. Int. J.   Syst. Assur. Eng. Manag. 2018;9(1):  131-138.  It has URL as follows:  <https://doi.org/10.1007/s13198-016-0490-7>   - Darvishi MT, Kheybari S, Khani F. A Numerical Solution of the Lax's 7th-order   KdV equation by Pseudospectral Method and Darvishi's Preconditioning. Int. J. Contemp. Math. Sci. 2007;2(22):  1097-1106.  It has URL as follows:  <https://www.researchgate.net/publication/269015023>   - Akinyemi L. q-Homotopy analysis method for solving the seventh-order   time-fractional Lax’s Korteweg-de Vries and Sawada Kotera equations. Comput Appl. Math. 2019;38:191.  It has URL as follows:  <https://doi.org/10.1007/s40314-019-0977-3>   - Siraj-ul-Islam, Aziz I, Sarler B. The numerical solution of second-order   boundary-value problems by collocation method with the Haar wavelets. Math.  Comput. Model. 2010;52:1577-1590.  It has DOI as follows:  doi:10.1016/j.camwa.2009.12.005   - No, there is no ethical or legal restriction. - It is okay for limited access. |
| 5. | We note that your manuscript is not formatted using one of PLOS ONE’s accepted file types. Please reattach your manuscript as one of the following file types: .doc, .docx, .rtf, or .tex (accompanied by a .pdf).  If your submission was prepared in LaTex, please submit your manuscript file in PDF format and attach your .tex file as “other.” | Our manuscript is written the template file of PLOS ONE given on the website.  I shall attach manuscript as .tex file.  The paper is written in Latex, and .tex file along with the pdf will be uploaded as other file. |

**Note**: It is confirmed that the information relevant to “Data Availability” is accurate. Please update my “Data Availability Statement” accordingly. Thank you.
